# Phylogenetic and Mutational Analysis of Lassa Virus Strains Isolated in Nigeria: Proposal for an In Silico Study

**DOI:** 10.2196/23015

**Published:** 2021-03-26

**Authors:** Daniel Kolawole, Hayatu Raji, Malachy Ifeanyi Okeke

**Affiliations:** 1 Department of Natural and Environmental Sciences American University of Nigeria Yola Nigeria

**Keywords:** Arenavirus, Bayesian phylogeny, epidemic, evolution, Lassa virus, Mammarenavirus, marker gene, molecular epidemiology, mutations, Nigeria

## Abstract

**Background:**

In 2018, the total number of Lassa fever cases in Nigeria was significantly higher than that observed in previous years. Hence, studies had attempted to determine the underlying cause. However, reports using phylogenetic methods to analyze this finding ruled out the emergence of potentially more transmissible Lassa virus strains or an increase in human-to-human viral transmission as the cause underlying the increase in cases. Two years later, the situation seems even worse as the number of confirmed cases has reached an all-time high according to situational reports released by the Nigerian Center for Disease Control.

**Objective:**

Considering the increasing trend of Lassa fever cases and related mortality, the major objective of this study is to map mutations within the genomes of Lassa virus isolates from 2018 and 2019 using the reference sequence available at the National Center for Biotechnology Information as a benchmark and compare them to the genomes of viruses isolated during 1969-2017. This study would also attempt to identify a viral marker gene for easier identification and grouping. Finally, the time-scaled evolution of Lassa virus in Nigeria will be reconstructed.

**Methods:**

After collecting the sequence data of Lassa virus isolates, Bayesian phylogenetic trees, a sequence identity matrix, and a single nucleotide polymorphism matrix will be generated using BEAST (version 2.6.2), Base-By-Base, and DIVEIN (a web-based tool for variant calling), respectively.

**Results:**

Mining and alignment of Lassa virus genome sequences have been completed, while mutational analysis and the reconstruction of time-scaled maximum clade credibility trees, congruence tests for inferred segments, and gene phylogeny analysis are ongoing.

**Conclusions:**

The findings of this study would further the current knowledge of the evolutionary history of the Lassa virus in Nigeria and would document the mutations in Nigerian isolates from 1969 to 2019.

**International Registered Report Identifier (IRRID):**

DERR1-10.2196/23015

## Introduction

Lassa fever, a viral hemorrhagic fever, is caused by Lassa virus. Viral hemorrhagic fevers are a group of viral illnesses that are characterized by damage to the vascular system; hence, they are described as being “hemorrhagic” [1]. Viral hemorrhagic fevers are caused by enveloped RNA viruses from 4 families: Arenaviridae, Bunyaviridae, Filoviridae, and Flaviviridae [1,2]. The major natural reservoirs of hemorrhagic viruses are usually rodents, fruit bats, and nonhuman primates [3].

Lassa virus is an ambisense RNA virus that belongs to family Arenaviridae and genus *Mammarenavirus*. There are 35 currently recognized viruses within this genus, which are classified into Old World and New World viruses, and Lassa virus is an Old World virus [4]. The Lassa virus genome contains 2 segments—the L (7.3 kb) and S (3.4 kb) segments—each encoding 2 proteins. The L segment encodes the viral RNA polymerase and zinc-binding proteins, while the S segment encodes the nucleoprotein and glycoprotein precursors [5]. Lassa fever is endemic to various regions of West Africa including Nigeria, Guinea, Sierra Leone, Liberia, Benin, Ghana, Côte d’Ivoire, Togo, and Mali.

Lassa virus is a very diverse group as multiple lineages have been inferred from numerous sequencing projects. The consensus seems to be that Lassa viruses can be categorized into four lineages: I, II, III, and IV. Lineages I, II, and III are endemic to Nigeria, while lineage IV is endemic to Guinea, Sierra Leone, Côte d’Ivoire, Mali, and Liberia [6]. However, other lineages of Lassa virus have been reported across West Africa. Strains V and VI have been proposed after sequencing of strains isolated from Côte d’Ivoire and Mali and from *Hylomyscus pamfi* rodents in Nigeria [7].

Owing to its zoonotic nature, humans can develop Lassa fever upon viral transmission on coming in contact with excreta, urine, or tissue of the reservoirs of the virus. The reservoirs for Lassa virus have been identified to be rodents of genus *Mastomys*, otherwise known as “Multimammate rats” [6,8]. Human-to-human transmission of Lassa virus is also possible most commonly through the nosocomial route when healthy individuals come in direct contact with medical instruments contaminated with Lassa virus or the blood, urine, feces, and other body secretions of a patient with Lassa fever [6,9].

**Figure 1:**
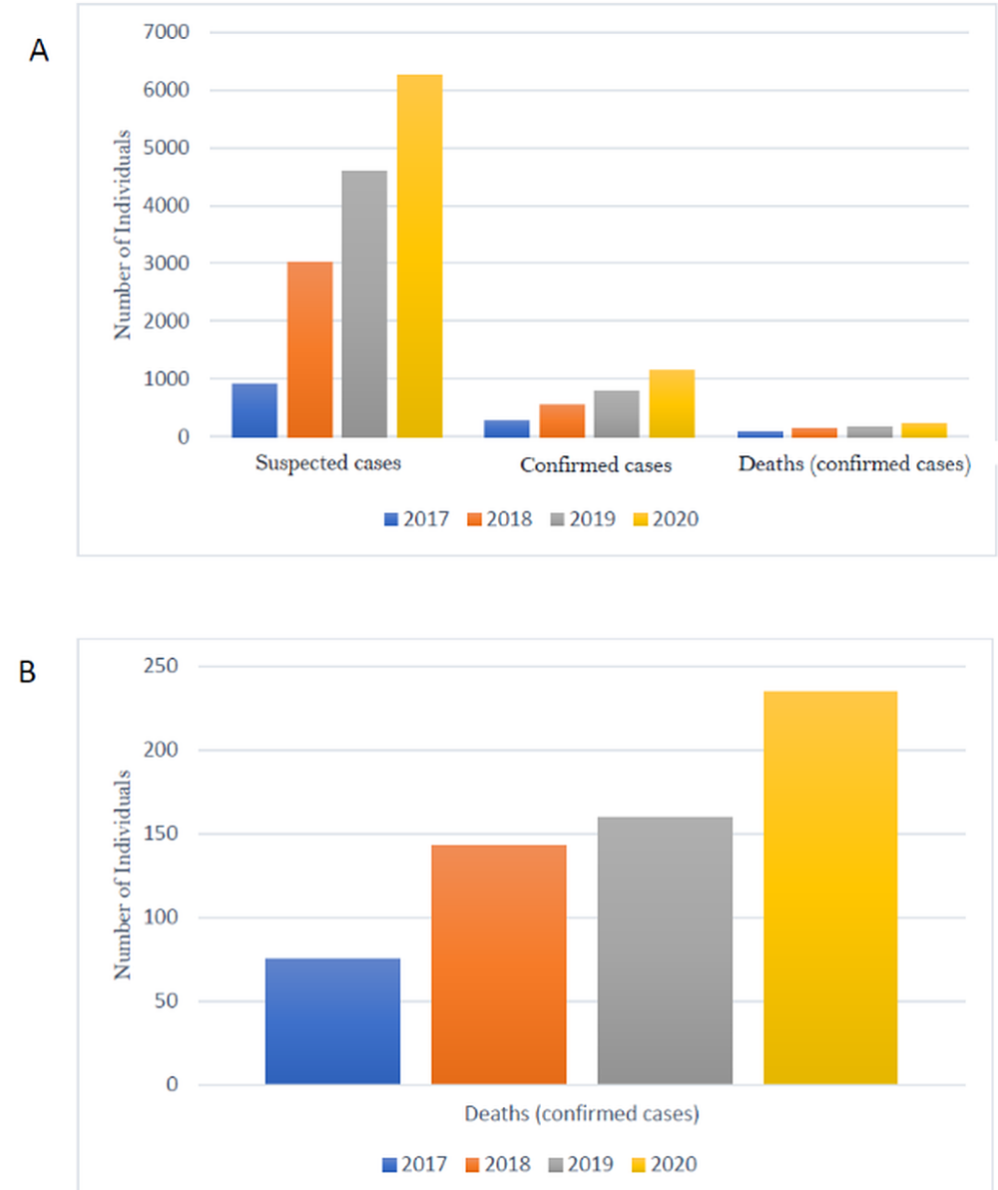
(A) Year-by-year trends in the epidemiology of Lassa fever in Nigeria showing total suspected cases, confirmed cases, and the death toll for confirmed cases as reported by the Nigerian Center for Disease Control as at week 46 of every year. (B) Confirmed deaths due to Lassa fever in Nigeria for period 2017 to 2020.

The number of Lassa fever cases was higher in the outbreak in the 2018 transmission season than in previous years in Nigeria [8,10]. Consequently, investigational studies were conducted to address concerns regarding the emergence of a more transmissible Lassa virus strain [8,10]. However, upon genome analysis, it was found that the viruses from the 2018 outbreak clustered with isolates from previous years; therefore, these viruses are believed to originate from the same pool of lineages known to circulate in Nigeria. Furthermore, it was determined that the upsurge in infection rates was not sustained through extensive human-to-human transmission, indicating that the epidemic was fueled by independent zoonotic events, thus dismissing concerns regarding a more transmissible strain [8,10]. Further, unpublished data in 2019 [11-13] are concurrent with the findings of Kafetzopoulou et al [8] in 2018. However, a worsening trend has been observed in subsequent years; with even more Lassa fever cases and related mortalities. Overall, the epidemic curve of Lassa fever in Nigeria has increased from 2017 till date (Figure 1). Therefore, we hypothesize that Lassa virus strains fueling the epidemic since 2017 and onwards are distinct from those isolated before 2017 in terms of small-scale mutations but not overall phylogenetic clustering patterns. Therefore, our proposed study would focus on determining the mutational profile of Lassa virus strains isolated in 2018 and 2019 and to compare them with isolates from previous years, identify gene trees that may serve as surrogates for species trees, and help reconstruct the time-scaled evolution of Lassa virus in Nigeria.

To our knowledge, no studies have reported a marker gene for Lassa virus in Nigeria. The identification of a gene or combination of genes that serve as surrogates for species trees would facilitate real-time monitoring of Lassa virus evolution and transmission and aid in routine diagnosis of Lassa fever. A demonstration of phylogenetic congruence between gene trees and the S and L segment trees would provide evidence supporting the utility of such a gene as a marker for Lassa virus whole-genome phylogeny.

There is a paucity of information regarding genome-wide mutational analysis of Lassa virus isolated in Nigeria in 1969-2019. Comparative mapping of mutations (indels and single nucleotide polymorphisms [SNPs]) in the Lassa virus genomes may provide mutation signatures of Lassa virus. Although the potential mutational differences between strains isolated in 2018-2019 and before 2018 (1969-2017) would not provide insights into the increased transmissibility of the virus in Nigeria in and after 2018, functional analysis of the identified mutations in future studies would provide valuable insights into the role of indels and SNPs (if any) in Lassa virus infections. Hence, *in silico* mapping of mutations in the virus genomes before future functional analysis of identified mutations would be suitable.

Although Ehichioya et al [14] constructed time-scaled maximum clade credibility trees for Lassa virus isolated in Nigeria, they did not include strains from 2019; they analyzed only S and L segments but did not include genes encoding the Z protein, L polymerase, nucleoprotein, and glycoprotein. Furthermore, they did not perform congruence tests to determine whether the S segment tree is congruent with the L segment tree. Our proposed study will address the aforementioned knowledge gaps in an attempt to provide an updated evolutionary insight into Lassa virus in Nigeria.

## Methods

### Sequence Collection, Phylogenetic Analysis, and Congruence Test

Lassa virus sequences would be collected from GitHub [15] following sequencing efforts through a collaboration between the Irrua Specialist Teaching Hospital, Institute for Lassa Fever Research and Control (Irrua, Edo State, Nigeria); the Bernhard-Nocht Institute for Tropical Medicine (Hamburg, Germany); the Rega Institute/KU Leuven (Leuven, Belgium); the ARTIC network; and Public Health England (PHE) (Salisbury, the United Kingdom). The collection includes sequences isolated from 1969 to 2019 and contains a total of 415 L segment sequences and 499 S segment sequences. The sequences, as seen in the GitHub repository [15], have their noncoding segments excised. Owing to resource limitations, a subset of sequences for the L and S segments would be drawn from the original set. Members of the subset would be selected to represent all major and minor clades within the Lassa virus population. After collection, the sequences would be aligned using MAFFT (version 7.450) using the default settings [16]. After alignment, Bayesian phylogenetic analysis would be performed using BEAST (version 2.6.2) [17]. The analysis would be performed using the strict clock and the coalescent constant population tree prior parameters. Custom priors would be added to check for the time to the most recent common ancestor for the 2018 and 2019 virus isolates. The tree file generated as output from Bayesian analysis would be used to construct a time-scaled maximum clade credibility tree by using TreeAnnotator (version 2.6.2) [17], and this tree would be visualized using FigTree (version 1.4.4) [18] where a timescale would be added with the specimen collection date for calibration. The aforementioned steps from alignment to phylogenetic analysis would be repeated for each gene. Genes can be extracted from each genome segment through the annotation provided by National Center for Biotechnology Information [19,20]. Finally, congruence tests between the gene and segment phylogenies would be conducted using the Kishino-Hasegawa, Shimodiara-Hasegawa, and approximately unbiased tests using IQ-TREE 2 [21,22] with default parameters.

### Sequence Similarity Matrix and Mutation Mapping

The percent identity matrix of both segments of the *Mammarenavirus* genome would be obtained using the Base-By-Base bioinformatics program [23]. Owing to the large number of sequences, the percent identity matrix would only be obtained for a small fraction of the entire set of sequences, which is highly representative of the general population.

The 2018 and 2019 isolates would be extracted from these gene sequence alignments. All gaps would be deleted to produce the raw order of bases in all isolates. Thereafter, all sequences would be translated in silico into amino acid sequences, and the reference sequence for each protein [24-27] would be added. After amino acid sequence alignment, the web-based tool DIVEIN [28] would be used to call out variable sites within all protein sequences, which can then be visualized in Microsoft Excel.

## Results

This study commenced in January 2020 as an obligatory senior undergraduate research project of the first author (DK) and will be completed in February 2021 with a thesis presentation and defense. Data mining and alignment of Lassa virus genome sequences have been completed. Phylogenetic and genomic analysis of aligned Lassa virus genome sequences are currently ongoing. A total of 133 L and 145 S segments from 1969 to 2019 were used for the genome-wide mutational and phylogenetic analyses (Table 1). Segment lengths upon multiple sequence alignment with gaps removed for the L and S segments and 4 genes are depicted in Table 2.

**Table 1:** Number of sequences of the L and S segments of the Lassa virus genome from 1969 to 2019 used in this study.

Year	Number of Lassa virus genome sequences	
	L segment	S segment
1969	1	1
1974	0	1
1976	1	1
1977	0	1
1981	1	2
1982	1	1
1999	1	1
2000	2	2
2003	1	1
2008	11	11
2009	12	12
2010	10	11
2011	12	10
2012	14	13
2013	4	8
2014	3	4
2015	3	5
2016	12	13
2017	3	3
2018	21	24
2019	20	20
Total	133	145

**Table 2:** Sequence length (nt) of aligned segment and gene sequences after gap removal.

Segment		Length of the aligned sequence (nt)
**L segment**		6809
	Z protein	297
	L polymerase	6512
**S segment**		3186
	Nucleoprotein	1710
	Glycoprotein	1476

## Discussion

Unlike previous reports, this proposed study will focus on determining SNPs and indel profiles of the 2018 and 2019 isolates of Lassa virus using the reference sequences as a benchmark for comparison. The identification of such SNPs and indels may further our understanding of the molecular factors contributing to the upsurge in Lassa fever cases in Nigeria in 2018 till date. SNP and indel identification may also help explain the limited instances of potential human-to-human transmission of Lassa fever as suggested in a previous report [10]. The identification of a marker gene that predicts whole-genome phylogeny can be useful in situations of limited resources. This marker gene can be sequenced and used for more rapid evolutionary analysis of Lassa virus. The inclusion of sequences from 2019 and congruence tests for tree topology in the reconstruction of a time-scaled maximum clade credibility tree will provide an updated and more robust analysis of Lassa virus evolution in Nigeria.

A major limitation of this proposed study is that the genome sequences of viruses isolated from rodent reservoirs during the 2018 outbreak are unavailable; hence, this study will not be able to examine whether the upsurge resulted from increased rodent-to-human transmission. A comparison of the genome sequences of Lassa virus isolated from rodent reservoirs with those of the 2018 and 2019 human isolates would reveal sequence signatures that may be associated with increased rodent-to-human transmission. Thus, viral adaptation for increased transmissibility may have occurred in the rodent reservoir rather than in humans. Extensive retrospective and prospective sampling of the Lassa virus in reservoir hosts is needed to examine our hypothesis of rodent-to-human transmission. Another limitation is that Lassa virus genome sequences from 2020 are not readily available.

The strengths of this proposed study include the public availability of Lassa virus genomes isolated during the 2018 and 2019 outbreaks, application of congruence tests to inferred phylogenetic trees, and mapping of mutations that may be masked by phylogenetic signals. Collectively, the strengths of this proposed study will more comprehensively elucidate the evolutionary history of Lassa virus in Nigeria.

## Abbreviations

SNP: single nucleotide polymorphism
